# Nanoscale Cathodoluminescence and Conductive Mode Scanning Electron Microscopy of van der Waals Heterostructures

**DOI:** 10.1021/acsnano.3c03261

**Published:** 2023-06-15

**Authors:** Hugh Ramsden, Soumya Sarkar, Yan Wang, Yiru Zhu, James Kerfoot, Evgeny M. Alexeev, Takashi Taniguchi, Kenji Watanabe, Sefaattin Tongay, Andrea C. Ferrari, Manish Chhowalla

**Affiliations:** † Department of Materials Science and Metallurgy, University of Cambridge, 27 Charles Babbage Road, Cambridge, CB3 0FS, United Kingdom; ‡ Cambridge Graphene Centre, University of Cambridge, 9 J. J. Thomson Avenue, Cambridge, CB3 0FA, United Kingdom; § Research Center for Materials Nanoarchitectonics, National Institute for Materials Science, 1-1 Namiki, Tsukuba 305-0044, Japan; ∥ Research Center for Electronic and Optical Materials, National Institute for Materials Science, 1-1 Namiki, Tsukuba 305-0044, Japan; ⊥ School for Engineering of Matter, Transport and Energy, Arizona State University, Tempe, Arizona 85287, United States

**Keywords:** layered materials, cathodoluminescence, nanoscale, scanning electron microscopy, transition metal dichalcogenides, van der Waals heterostructures

## Abstract

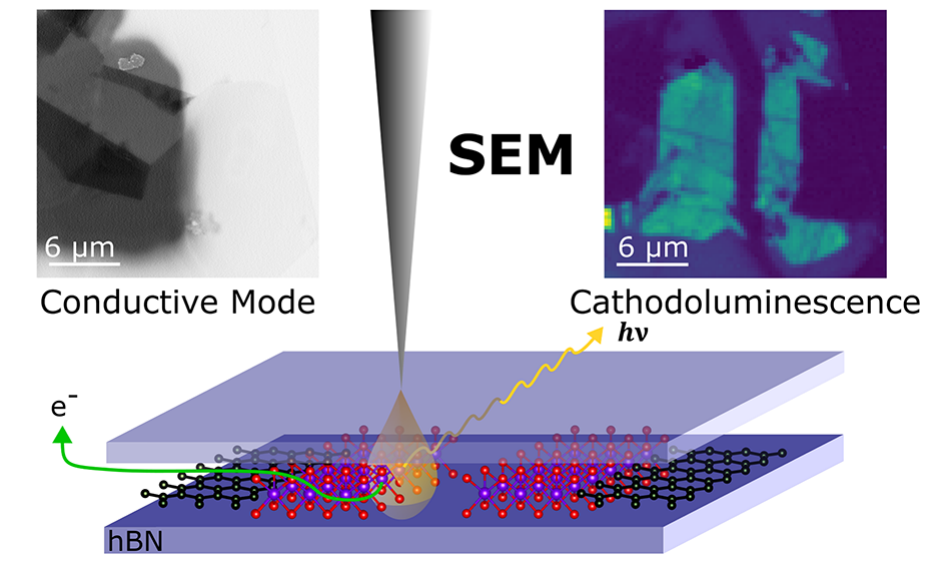

van der Waals heterostructures (vdW-HSs) integrate dissimilar materials to form complex devices. These rely on the manipulation of charges at multiple interfaces. However, at present, submicrometer variations in strain, doping, or electrical breakages may exist undetected within a device, adversely affecting macroscale performance. Here, we use conductive mode and cathodoluminescence scanning electron microscopy (CM-SEM and SEM-CL) to investigate these phenomena. As a model system, we use a monolayer WSe_2_ (1L-WSe_2_) encapsulated in hexagonal boron nitride (hBN). CM-SEM allows for quantification of the flow of electrons during the SEM measurements. During electron irradiation at 5 keV, up to 70% of beam electrons are deposited into the vdW-HS and can subsequently migrate to the 1L-WSe_2_. This accumulation of charge leads to dynamic doping of 1L-WSe_2_, reducing its CL efficiency by up to 30% over 30 s. By providing a path for excess electrons to leave the sample, near full restoration of the initial CL signal can be achieved. These results indicate that the trapping of charges in vdW-HSs during electron irradiation must be considered, in order to obtain and maintain optimal performance of vdW-HS devices during processes such as e-beam lithography or SEM. Thus, CM-SEM and SEM-CL form a toolkit through which nanoscale characterization of vdW-HS devices can be performed, allowing electrical and optical properties to be correlated.

Monolayer (1L) transition metal dichalcogenides (TMDs) are interesting for optoelectronic^1^ and electronic^2^ applications due to their direct band gap,^3^ high room temperature mobility^4^ (∼200 cm^2^ V^–1^ s^–1^), strong excitonic binding energy^5^ (>200 meV), and valley polarization.^6^ These properties are sensitive to external factors, such as charge traps,^7^ impurities,^8^ and strain.^9,10^ To assess their influence at nanometer length scales, tip-enhanced methods can be used.^11−13^ However, to obtain optimal properties such as low transistor hysteresis^14^ or narrow luminescence line widths,^15^ encapsulation of TMDs in hBN is often necessary, as it provides an atomically smooth substrate and a uniform dielectric environment.^16^ This presents an issue, as the near-field phenomena that tip-enhanced spectroscopy relies on cannot be achieved in structures buried under hBN.^17,18^

Cathodoluminescence (CL) has emerged as a route to study TMDs and encapsulated vdW-HSs at nanometer length scales.^19−21^ CL is analogous to photoluminescence (PL), except the excitation is provided by an electron beam.^22^ SEM-CL can reach spatial resolutions of <100 nm.^19^ Different depths within the sample can also be probed by tuning the energy of the electron beam.^23^ Through CL, nanoscale variations in strain^20^ and doping^21,24^ of hBN encapsulated TMDs have been assessed through correlation with their optical emission.^20,21,24^ These results have shown that nanoscale heterogeneities in doping and strain exist in TMD-based devices.^20,21,24^

The performance of vdW-HSs is influenced by the efficiency of electron flow across interfaces between the constituent materials.^25,26^ Conductive mode scanning electron microscopy (CM-SEM) measurements can be used to study the flow of electrons across vdW-HS interfaces at the nanoscale by mapping pathways of injected electrons.^27,28^ This method has been used for failure analysis of semiconductor chips, by allowing breakages in submicrometer power rails to be visualized.^27,29^

Here, we use SEM-CL and CM-SEM to characterize hBN/1L-WSe_2_/hBN devices. Through CL we assess nanoscale strain variations in the semiconducting 1L-WSe_2_ arising from the fabrication process. Through CM-SEM, we study the flow of electrons deposited into the vdW-HS during the measurement. Simultaneous acquisition of SEM-CL and CM-SEM correlates the electron flow with luminescence.

## Results and Discussion

### Assessment of vdW-HS Quality through SEM-CL

**Figure 1:**
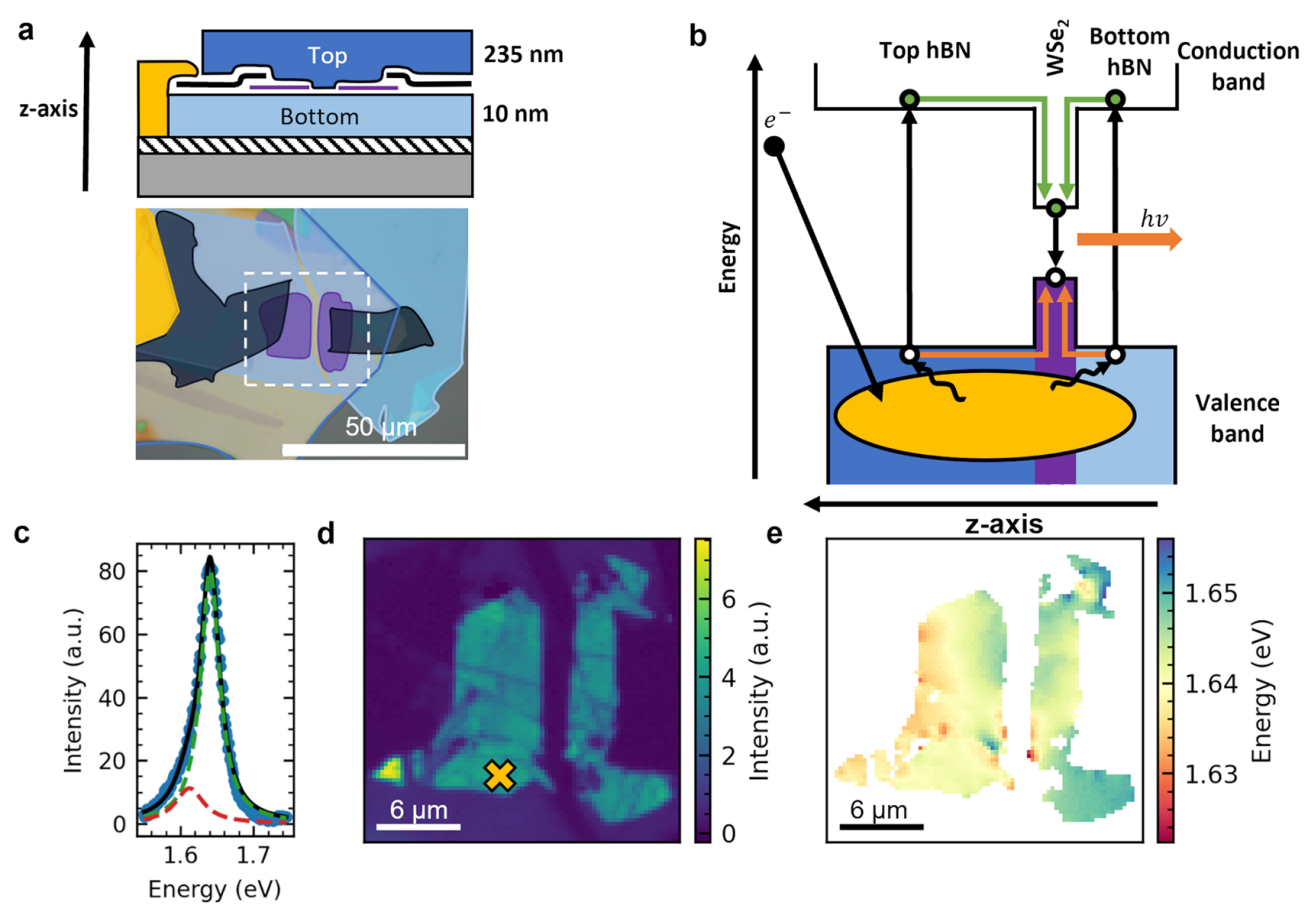
a) Top: device cross section. 1L-WSe_2_, is contacted by FLG and encapsulated in hBN. One side of FLG is contacted by an Au electrode. Bottom: Optical microscopy image of the device with each material highlighted. The area outlined with a white dashed square is analyzed further via SEM. b) Illustration of the light generation process during CL. The electron beam impinges on the vdW-HS and generates a large number of electron–hole pairs within the interaction volume shown in yellow. These electron–hole pairs diffuse through the hBN and transfer into the 1L-WSe_2_. Finally, the electron–hole pairs recombine, resulting in the generation of light. c) CL spectrum from 1L-WSe_2_, taken from the pixel indicated by a yellow cross in panel (d). The peak is fitted well by a bi-Lorentzian fit, shown as a black line. Each component corresponds to luminescence from excitons and trions,^30^ shown in dashed blue and red lines, respectively. d) Pixel map of integrated CL counts over spectral range of the 1L-WSe_2_ peak (710–800 nm). The green and yellow regions indicate where the CL from 1L-WSe_2_ is bright. e) Pixel map of exciton energy extracted from pixel-by-pixel Lorentzian luminescence fits of the data in panel (d). Shifts in the exciton energy are an indication of submicrometer strain variations or other inhomogeneities.

Figure 1a shows an annotated optical microscope image of a representative device. Figure 1b illustrates how CL occurs using the band energy diagram for 1L-WSe_2_ encapsulated in hBN. The electron beam generates electrons and holes in hBN within the interaction volume, shown in yellow in Figure 1b. These then diffuse and transfer into 1L-WSe_2_, remaining trapped there, due to the Type I band alignment between 1L-WSe_2_ and hBN,^31,32^ then recombining, giving rise to CL.^20^ Previous works have shown^19,20^ in 1L-WSe_2_ without hBN encapsulation, the interaction of the electron beam with the sample is insufficient to obtain a detectable signal (also see Section S1 of Supporting Information).^33^ Thus, encapsulation in hBN is essential for CL measurements, as it effectively increases the interaction volume between electron beam and 1L-WSe_2_ by capturing excitons generated in the thicker surrounding hBN.^34^

A typical CL spectrum of 1L-WSe_2_ is shown in Figure 1c. We fit this with a bi-Lorentzian curve, shown in black. The main Lorentzian component corresponds to the A exciton peak (shown green), centered at 1.641 eV.^35^ The secondary Lorentzian component corresponds to the trion peak (shown red), at 1.612 eV, indicating a trion binding energy of ∼28 meV, in good agreement with previous reports.^3,30,36,37^Figure 1d is a spatial color map of integrated CL intensity around the 1L-WSe_2_ A exciton peak. Green and yellow pixels indicate regions where the emission from 1L-WSe_2_ is the strongest. No emission is seen from regions in contact with FLG, due to quenching of excited carriers.^38^

In addition to variations in the strength of CL intensity in Figure 1d, variations in the A exciton energy (extracted from Lorentzian fitting of the A exciton peak at pixels with an integrated intensity of >2400 counts) are also observed in Figure 1e. A number of external factors including temperature,^30,39^ local dielectric environment^40,41^ and strain^10,42^ influence the emission energy of excitons in 1L-TMDs. We discount heating effects as the cause of the variation of the CL peak position, since the thermal conductivity of the sample should not vary sufficiently across the map to yield large CL energy shifts. We estimate a maximum red shift of ∼21 meV upon heating from the ∼53 K extracted in Table S1 due to the incident electron beam.^30^ This is further confirmed by the absence of significant spectral wandering upon activation of the beam, as shown in Figure 3f and discussed later. Variations in dielectric environment influence the exciton binding energies of 1L-TMDs. Using the model of ref (40), we estimate a decrease in exciton binding energy of 1L-WSe_2_ of ∼70 meV between full hBN encapsulation (dielectric constant: ε = 4.5 on the top and bottom) and partial hBN encapsulation (ε = 4.5 on the top and ε = 1 on the bottom). Such an arrangement may be expected over localized features, such as bubbles, though our fabrication approach is used to remove many such features.^43^ However, as intimate contact between hBN and 1L-WSe_2_ is essential to facilitate efficient transfer of electron–hole pairs into the 1L-WSe_2_,^8^ regions where 1L-WSe_2_ is not in good contact with hBN will appear dim.^8^ In Section S6 of the Supporting Information we show that there is no correlation between peak intensity and peak energy. Therefore, we believe by only performing peak position analysis on regions of the sample with bright emission, we exclude dielectric environment variations as a cause for CL emission shifts. Nonetheless, CL studies ought to consider the effect of dielectric environment. CL would be a powerful tool to study this in TMDs in close proximity to dielectric nanostructures.^44^

We note that in the CL spectra tensile strain would give a red shift and compressive strain would give a blue shift.^10^ We therefore ascribe features such as the red shift in CL emission close to the left FLG electrode to be a region of tensile strain, which may indicate localized mechanical stresses imparted during the transfer process. In Section S2 of the Supporting Information we compare the strain variations detected through CL (standard deviation of ∼0.05%) to those estimated through Raman spectroscopy (a variation of ∼0.13%) and find good agreement, further asserting the attribution of peak variations to strain. The ability to detect nanoscale regions of localized strain is important as it may influence device performance, for example, by giving rise to undesirable inhomogeneous broadening of the luminescence or influencing the carrier mobility.^45,46^

### Studying Electron Flow in vdW-HS Devices through CM-SEM

**Figure 2:**
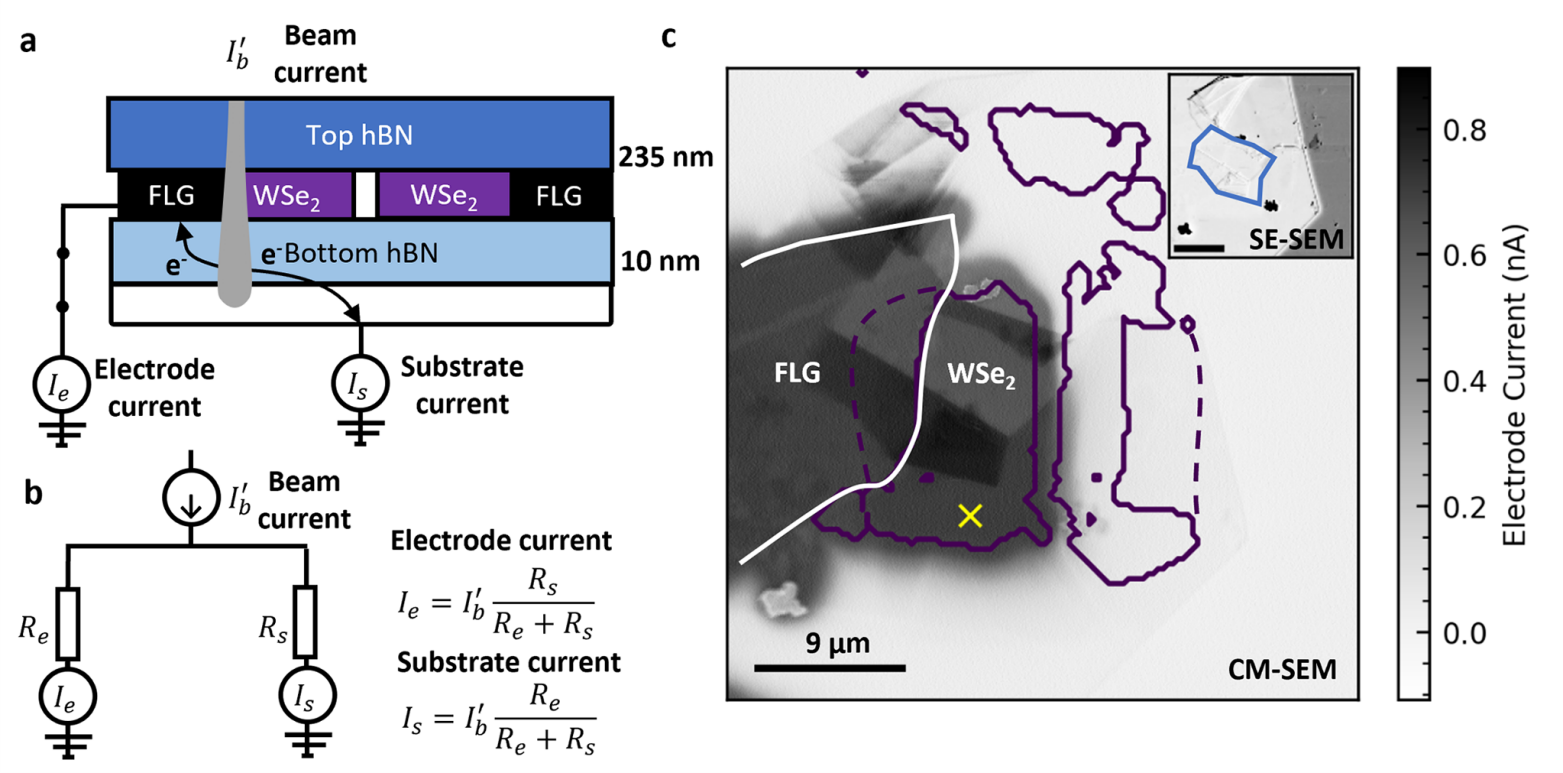
a) Scheme of RCI measurement. The beam injects electrons into the vdW-HS. These can take a path to ground via the substrate or FLG electrode, detected as currents *I*_*s*_ and *I*_*e*_, respectively. b) Equivalent circuit diagram for RCI measurement. The paths to the ground via the FLG electrode or via the substrate act like a current divider. Hence a current through the electrode (*I*_*e*_) indicates that this path is the lowest resistance route for electrons to leave the sample. c) Simultaneous RCI and CL map of vdW-HS. The grayscale image shows a pixel-by-pixel map of *I*_*e*_ (note: positive currents represent electrons leaving the sample). The dark gray region is where the device current is highest and corresponds to the grounded FLG electrode and electrically connected 1L-WSe_2_. The solid purple line outlines regions where a strong CL signal from 1L-WSe_2_ is measured, and the dashed purple line outlines the region of 1L-WSe_2_ underneath FLG. The white line outlines the region where the FLG electrode is present. While bright CL is seen from the 1L-WSe_2_ on the right-hand of the device, no RCI current is measured, indicating these regions are not electrically connected to the grounded FLG electrode. A secondary electron (SE) image is simultaneously acquired during RCI and CL mapping, as shown in the inset in which a folded piece of hBN on the surface is outlined in blue. In this SE image, the buried 1L-WSe_2_ and FLG cannot be resolved.

We used CM-SEM to probe the flow of charges during electron irradiation. We focus on the part of the sample schematically illustrated in Figure 2a. As the electron beam can penetrate the vdW-HS, this technique is akin to inserting a nanometer-scale electronic probe into a buried device. Figure 2a illustrates the current flow between FLG contacted with 1L-WSe_2_ and substrate. As indicated in Figure 2b, for any electrons injected into the sample, this circuit acts like a current divider. If a higher current is measured from the electrode (*I*_*e*_) compared to the substrate (*I*_*s*_), then this is an indication that electrons prefer to take this path as it provides a lower resistance route to ground. This technique is known as resistive contrast imaging (RCI).^27,29,47^

We scan a 6 keV, 1 nA beam over the sample, measuring the substrate current as a function of beam position to build a pixel-by-pixel map of electron current, as shown in Figure 2c. We also superimpose an outline of the region where CL from 1L-WSe_2_ is seen. Examining the RCI map, an electrode current of ∼0.7 nA is measured at the left FLG electrode connected to 1L-WSe_2_, and there is almost zero electrode current elsewhere. This demonstrates that electrons are injected into 1L-WSe_2_ and are conducted to FLG.

There is no significant difference in electrode current between FLG and 1L-WSe_2_, suggesting that the contact resistance between these materials is negligible. The ∼0.7 nA current measured at the 1L-WSe_2_ and electrode suggests that up to 70% of beam electrons enter 1L-WSe_2_ during irradiation of 1 nA at 6 keV.

A secondary electron (SE) image acquired simultaneously with the RCI map is shown in the inset of Figure 2c. In this, some regions of hBN that have folded over on the surface can be seen, outlined in blue. These folded regions give rise to nanometer variations in sample thickness that are reflected in the RCI signal. In the SE image, no clear signal from the buried FLG is evident. The region of grounded FLG can be resolved through RCI, despite being buried under hBN. The lack of electrical connection between left and right pieces of 1L-WSe_2_ is very clear, as no electrode current is seen from the right (floating) 1L-WSe_2_. A further discussion of the edge response of RCI in these samples is provided in Section S3 of the Supporting Information. This demonstrates that failure analysis through CM-SEM is also possible.

**Figure 3:**
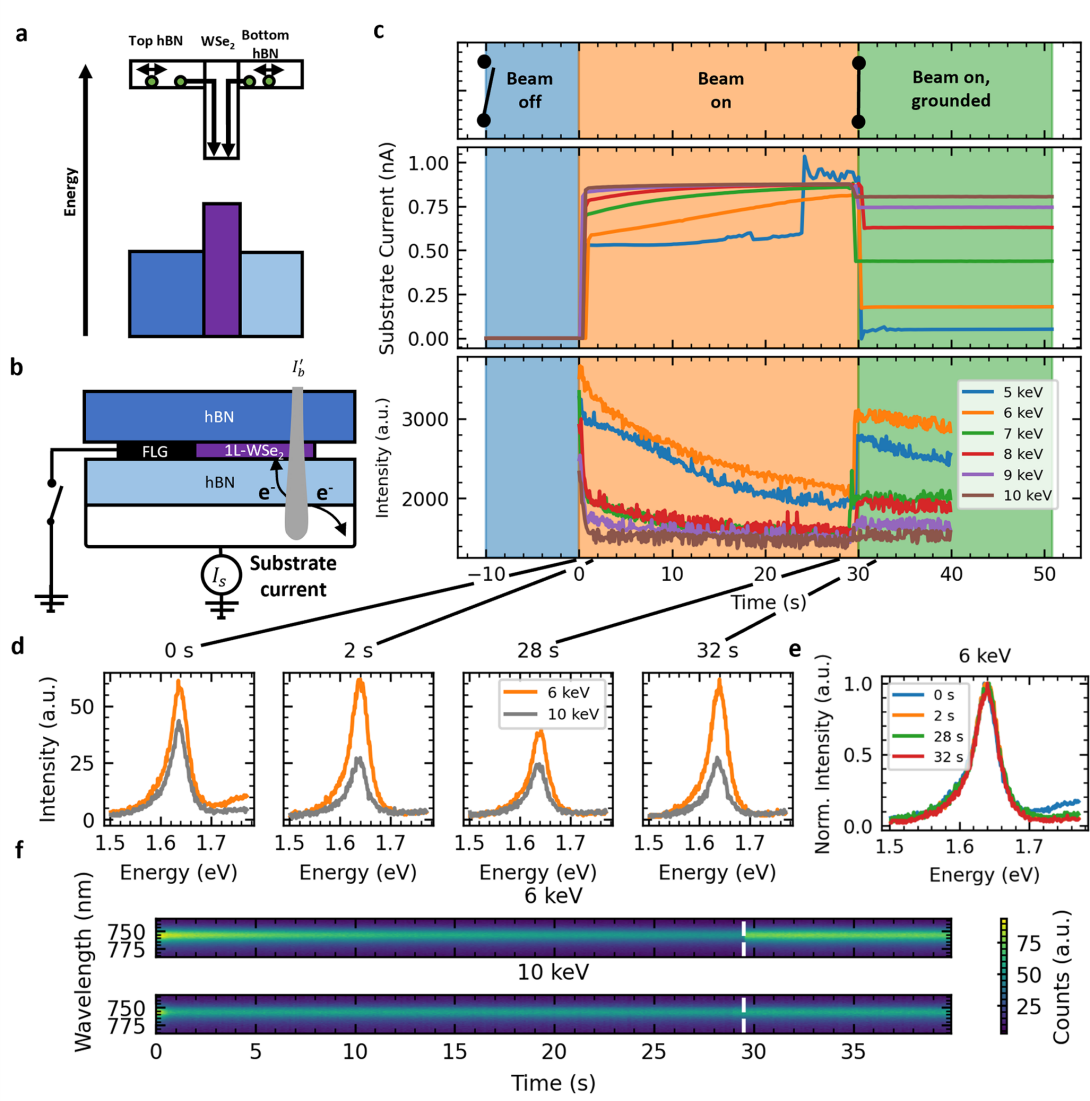
a) Illustration of trapping of injected electrons by the quantum well formed by the Type I band alignment between hBN and 1L-WSe_2_. b) Schematic setup used for dynamic CM-SEM measurements. The FLG electrode is connected and disconnected to the ground by using a switch. The current leaving the device through the substrate is measured. c) Time evolution of sample current and CL intensity at a fixed position for various acceleration voltages. The switch in panel (b) is kept open, the beam is kept off from −10 to 0 s, and then the beam is turned on for 40 s. At 30 s, the switch is closed and a path to ground via the electrode is established. The substrate current is measured as shown in panel (b). The CL intensity is the integrated intensity of the CL spectra at each time interval. While the beam is on and the path to ground via the FLG electrode is broken, the sample current gradually increases and the CL intensity decreases. d) CL spectra at various time slices for both 6 and 10 keV beam energies. After 2 s, the CL at 10 keV is significantly more stable than that at 6 keV. e) Normalized 6 keV CL spectra from time slices in panel (d). No change in line shape is seen during measurement. f) Heatmap showing the spectral shape for 6 and 10 keV energies during irradiation. No spectral wandering is seen. The approximate time at which the switch is closed is indicated by the white dashed line. After this point, the CL intensity at 6 keV rises significantly.

Figure 2c confirms that electrons deposited on hBN can transfer to encapsulated 1L-WSe_2_. Figure 3a illustrates the band structure of a sample with no grounding electrode, i.e., a device structure often used in optoelectronic studies.^20,48^Figure 3a shows that, for a sample with this structure, the Type I band alignment between 1L-WSe_2_ and hBN will not only trap transferred excitons but also trap electrons, leading to charge accumulation. This may influence the optical and electronic properties.

### Dynamic Electron-Sample Interaction Effects

As the sample is under constant irradiation, the trapping of charges may give rise to dynamic effects. To investigate this trapping of charge, we utilize the setup in Figure 3b, with a switch added between FLG electrode and ground. This means we can isolate the 1L-WSe_2_ from the ground by opening and closing the switch. We then collect time dependent CL and CM-SEM data, as shown in Figure 3c.

In Figure 3c, over the first 10 s, the beam is switched off, and the FLG electrode is disconnected from the ground. Hence, the substrate current is zero and no CL is seen. At 0 s, we switch the beam on, at which point we see a sharp rise in the substrate current accompanied by the detection of CL from 1L-WSe_2_. As the irradiation continues, the CL intensity decreases.

This decrease in CL intensity is evidence of charge accumulation in the 1L-WSe_2_. When the FLG electrode is reconnected to ground, any accumulated charge can leave the device, thus the CL intensity is largely restored to the level initially seen during irradiation. Individual spectra for 6 and 10 keV during the first measurement (0 s), after initial stabilization (2 s), near point of maximum charging (28 s), and after restoration of ground (32 s) are in Figure 3d.

Comparing the 6 keV spectra at 28 and 32 s, we see that the brightest CL signals can be collected over long (>5 s) acquisition times by ensuring the sample is grounded. In Figure 3e we overlay the normalized CL spectra from Figure 3d. Examining the line-shape, we see it does not significantly vary during measurements. Previous works have shown that changes to the carrier concentration can modulate PL spectral contributions from charged excitons (trions).^3,36,37^ Therefore, the observed accumulation of charge in 1L-WSe_2_ may be expected to cause variations in the CL line-shape. However, the line-shape of emission from WSe_2_ is not affected by charge doping, as suggested by gate-dependent PL measurements shown in Figure S4b, where a similar reduction in PL peak intensity (∼25%) can be achieved through electrostatic doping. This suggests that the trion contribution does not change under these conditions.

Figure 3f plots the heatmap of each CL spectrum collected during the irradiation. No significant spectral wandering is seen for both 6 and 10 keV, showing that charging and doping effects can be neglected when using the peak position to extract an estimate for the strain in 1L-WSe_2_. This is not unexpected as the exciton energy in 1L-WSe_2_ is independent of carrier concentration.^3^ Conversely, the exciton binding energy is sensitive to doping in 1L-MoS_2_.^49^ As such, when CL is used to measure strain in MoS_2_ heterostructures, mitigating charging through grounding may be crucial.

A large decline in intensity over the first 2 s for 10 keV is also seen for voltages of >6 keV. This could be related to hole generation at the sample surface due to secondary electron emission, which may give rise to millisecond dynamic effects.^50,51^

**Figure 4:**
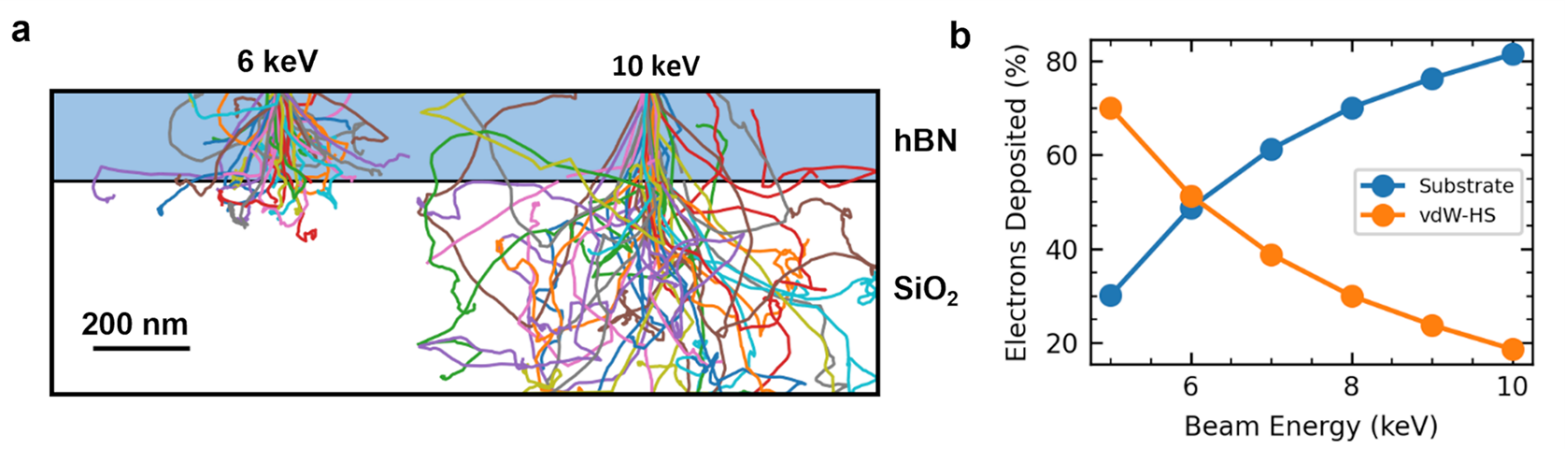
a) Monte Carlo predictions for the trajectory of 6 and 10 keV electrons through 245 nm hBN. Each line represents the path of an individual electron. For 6 keV, ∼50% of electrons come to rest (are deposited) within the hBN. Whereas, for 10 keV, ∼80% of electrons pass through and come to rest in the substrate. b) Monte Carlo predictions for the fraction of beam electrons deposited into the vdW-HS. As the acceleration voltage increases from 5 to 10 keV, significantly fewer electrons are deposited into the vdW-HS (from 70% to 20%).

The enhancement upon reconnection of the ground path displays a strong voltage dependence. To better understand the influence of beam energy, we performed Monte Carlo simulations of the interaction of the electron beam with our devices. We make use of the Nebula Monte Carlo simulation package,^52^ with which we simulate the path of 30,000 electrons through 245 nm of hBN on a deep SiO_2_ substrate (see Section S8 of the Supporting Information for more details). Figure 4a illustrates the simulated path of beam 6 and 10 keV electrons for the sample geometry used in this work. At higher acceleration voltages, more of beam electrons pass through the hBN and ultimately come to rest outside the vdW-HS. We then perform statistical analysis of the final position of electrons deposited into the sample. Figure 4b plots the percentage of beam electrons that come to rest within the vdW-HS versus the substrate over the acceleration voltages used in this study. Figure 4b shows that as the acceleration voltage increases from 5 to 10 keV, significantly fewer electrons are deposited into the vdW-HS (from 70% to 20%). Due to the quantum well structure in Figure 3a, electrons deposited into the vdW-HS may be trapped and accumulate in 1L-WSe_2_.

### Quantification of Accumulated Charge

**Figure 5:**
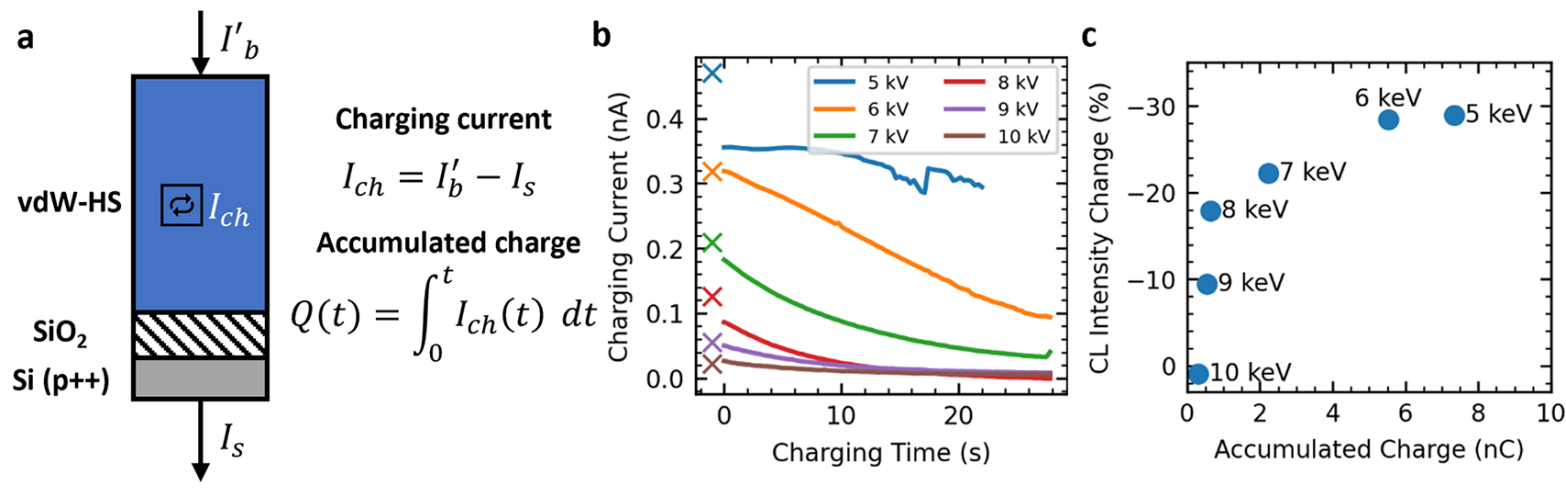
a) Illustration of charges entering and leaving vdW-HS during irradiation. *I*_*b*_^′^ is deposited into the sample and *I*_*s*_ leaves the sample. From the conservation of charge, any discrepancy between these values implies an amount of charge stored in the sample per unit time: *I*_*ch*_. This will cause vdW-HS to charge up. Integrating over *I*_*ch*_ allows the total charge accumulated in the vdW-HS to be calculated. b) Variation in charging current while the sample is irradiated (orange region, Figure 3c). This is the discrepancy between electrons into the sample and out. The initial charging currents predicted by Monte Carlo are plotted as crosses, with good agreement with experiments. In all cases, the charging current decreases over time. The lowest acceleration voltages correspond to the highest charging currents. c) Plot of CL intensity change after charging versus accumulated charge calculated from data in (b). The highest accumulated charges correspond to the highest CL intensity decrease.

We now show that the acceleration voltage tunes the amount of charge stored in 1L-WSe_2_, and we show how this influences the luminescence. The top of Figure 5a illustrates the currents that enter the sample, the beam current *I*′_*b*_, and exit the sample, the substrate current *I*_*s*_. From the conservation of charge, any discrepancies between these values imply storage of charge within the vdW-HS: *I*_*ch*_ = *I*_*b*_^′^ – *I*_*s*_. Figure 5b shows the variation in *I*_*ch*_ as the sample is irradiated. When the electrode is disconnected, carriers injected into hBN cannot travel to the ground, as they are trapped there by the band offset with SiO_2_, as illustrated in Figure S6.

Hence, the substrate current can arise only from electrons deposited into SiO_2_ and underlying Si. While electrical injection of carriers into SiO_2_ is difficult due to its large bandgap,^53^ the electron beam has sufficient energy to directly excite carriers.^54^ Furthermore, at room temperature, the mobility of excited electrons in SiO_2_ is sufficient for them to travel through SiO_2_.^54^

Figure 5b shows that the magnitude of the initial charging current is strongly dependent on the acceleration voltage, with higher voltages giving lower currents since higher voltages deposit less charge in the vdW-HS, as for Figure 4b. These values are in good agreement with *I*_*ch*_ predicted through Monte Carlo simulations (see Supporting Information Section S9 for more details and Figure 5b).

The charging current decreases with time for all acceleration voltages due to 2 competing charging and discharging processes. We expect that initially all electrons entering the vdW-HS will be stored there. However, as electrons accumulate, discharging increases. Eventually, the current entering the vdW-HS balances the current leaking out, and the two reach equilibrium. This is reflected in the data in Figure 5b. The charging current decreases over time, appearing to approach an asymptote. As illustrated in Figure 3a, without a grounding electrode the charge stored in 1L-WSe_2_ is trapped there by the quantum well. This could mean that accumulated charge in the 1L-WSe_2_ could persist, leading to semipermanent doping.^55,56^

The integration of the charging current over time gives the total charge, *Q*, accumulated within the device. Figure 5c plots the accumulated charge and corresponding decrease in CL intensity, taken as the percentage difference in CL intensity at maximum charging (28 s) compared to that after grounding (32 s), from Figure 3c. From this we can see that lower acceleration voltages correspond to larger charge accumulations.

The higher accumulation of charge for 5 keV may explain the discontinuity seen for 5 keV in Figure 5b. This may be caused by the breakdown of the SiO_2_ dielectric. This may also explain the negligible contact resistance observed in Figure 2c. Ref (57) reported that sufficient doping through ionic liquid gating can significantly lower the contact resistance for FLG contacts.^57^ Electron beam doping may have a similar effect. We also observe that the decrease in CL intensity is larger for higher charge accumulations. Ref (58) reported that accumulation of charge outside of the neutrality point in 1L-WSe_2_ leads to a decrease in luminescence quantum efficiency. This change is attributed to doping-induced effects, such as increases in Auger-like nonradiative recombination.^59^ As such, when doping by the beam is higher, the decrease in the CL intensity is larger.

The rate of charge accumulation can also be controlled through the control of beam parameters. To aid in the optimization of measurement conditions, Figure S8 presents the charging current for hBN based vdW-HSs during irradiation for various sample thicknesses.

## Conclusions

We used SEM-CL and CM-SEM to characterize buried vdW-HS devices. Through SEM-CL we uncovered nanoscale variations in strain arising from the fabrication process. We then showed that CM-SEM can be used to spatially map the flow of carriers. We detected electrons deposited into hBN and then transferred into the encapsulated TMD. We also observed dynamic doping of 1L-WSe_2_ when electrons accumulate in the sample, which reduces CL intensity, with the rate of doping controlled by tuning the beam acceleration voltage. By grounding the encapsulated 1L-WSe_2_, charge accumulation can be mitigated to achieve a stable device performance. These results have implications for the optimization of CL intensity for vdW-HS-based devices. If lower acceleration voltages are utilized, due to the greater interaction volume of excited carriers within the vdW-HS, then a larger CL intensity can be achieved.^20^ However, this volume is also accompanied by greater excess charge deposition. Hence, providing a route for excess charge to easily leave the device is essential for the stability and maximization of CL.

Thus, unintentional doping of samples during e-beam lithography and SEM imaging should be considered. As vdW-HS devices become more complex,^60,61^ the ability to probe spatial variations in samples, both in and out of plane, will become more important. SEM-CL and CM-SEM could be powerful ways to meet this requirement.

## Methods

### Device Fabrication

Bulk hBN crystals are prepared by high-pressure Ba-BN synthesis^62^ bulk WSe_2_ is synthesized using flux zone growth^63^ and graphite crystals are sourced from NGS. Flakes are then prepared via micromechanical cleavage.^64^ Multilayer hBN (∼10 nm and ∼235 nm for bottom and top layers, as determined by AFM measurements, see Figure S9) flakes on 90 nm SiO_2_/Si are identified using optical microscopy, and the thickness of the top flake is chosen to improve optical contrast of the buried vdW-HS interfaces and offer a greater volume with which incident electrons interact. FLG flakes (*N* > 3 layers) on 285 nm SiO_2_/Si are identified, and their thicknesses estimated using optical microscopy^65^ and Raman spectroscopy. 1L-WSe_2_ flakes on A8-PMMA are identified using optical and PL microscopy. A8-PMMA substrates are used to prepare 1L-WSe_2_ in order to increase the size and yield of suitable flakes compared to SiO_2_. 90 nm SiO_2_/Si is used for hBN exfoliation to increase the optical contrast for TMDs, while 285 nm SiO_2_/Si is used for FLG, since this maximizes contrast.^65^

vdW-HSs are then prepared via a dry transfer process.^43^ A polycarbonate stamp is placed on a micromanipulator stage coupled to an optical microscope and used to align and pick up the top ∼235 nm thick hBN, left FLG, right FLG, 1L-WSe_2_, and bottom ∼10 nm thick hBN. To form the final device, the vdW-HS is then brought into contact with 285 nm SiO_2_/Si on a heated stage at 165 °C before increasing the temperature to 180 °C to melt the polycarbonate and promote the mechanical removal of trapped contaminants.^43^ The polycarbonate film is washed away via immersion in chloroform and ethanol. A Cr/Au electrode on FLG is defined by using direct write optical lithography (Microtech). Characterization of the as-fabricated device is provided in Section S2 of the Supporting Information.

### Cathodoluminescence

CL measurements are performed using an Attolight Allalin 4027 Chronos CL-SEM system. CL spectra are recorded using a iHR320 spectrometer (focal length 320 mm, 150 gratings per mm blazed at 500 nm, 700 μm entrance slit) with an Andor Netwton Bx-DD charge couple device (CCD). The readout rate is fixed at 50 kHz with an acquisition time of 100 ms. Background subtraction of the data is performed prior to analysis. All measurements are taken at room temperature. Fitting of CL data is done using Lumispy and Hyperspy Python libraries.^66,67^ CL spectra were fitted with two Lorentzian components. For the fitting used in Figure 1e, the exciton-trion separation was fixed at 28 meV, in accordance with the fit in Figure 1c, which was left free. The estimated standard deviations in exciton energy at each pixel are all below 1 meV, with the majority less than 0.5 meV.

### Conductive Mode Scanning Electron Microscopy Mapping

CM-SEM mapping is performed using a Femto DLPCA-200 variable gain low noise current amplifier in low speed (100 Hz) DC mode with an amplification ratio of 10^11^ V/A. For all CM-SEM measurements, the beam currents are tuned to 1.00 nA through a Faraday cup integrated in the CM-SEM sample holder to ensure precise and accurate current readouts. A dwell time of 1 ms is used to minimize capacitive effects.

### Time-Resolved Cathodoluminescence and Conductive Mode Scanning Electron Microscopy

All measurements are taken at a fixed position on the sample, indicated by the yellow cross in Figure 2c. This position is aligned by using SE features on the sample surface. Successive CL spectra are collected over 100 ms acquisition times. EBAC measurements are taken using a Keithley 6485 Picoammeter with 300 ms integration time.

### Monte Carlo Simulations

Monte Carlo simulations are performed using the Nebula Monte Carlo simulation tool.^52^ Primary beams are simulated using a Gaussian profile with full width at half-maximum of 5 nm. For the data presented in Figures 4b and S7, 30,000 primary electrons are used, and 5000 are used for Figure S8. Nebula produces an output of every electron collision and detection event. This output is analyzed using a custom Python code; see Supporting Information Section S8 for details.

### Gate-Dependent PL Measurements

Gate-dependent PL measurements were performed at room temperature using a home-built stage connected to a Keithley 2400 source meter in a Horiba LabRAM HR Evolution confocal spectrometer. The sample was excited with a 532 nm laser, and its power was kept below 10 μW.

### Atomic Force Microscopy

Atomic force microscopy measurements were performed using the Bruker Dimension Icon Pro system in tapping mode with a 0.7 Hz scan rate.

## Supporting Information Available

Comparison of CL signal without hBN encapsulation; characterization of as-fabricated device; comparison of spatial resolution of RCI and CL; discussion of beam induced heating of sample; discussion of CL line-shape; assessment of dielectric environment on emission energy; band diagram of vdW-HS; details of Monte Carlo simulation methods; estimation of charge deposition vs beam energy and sample thickness; AFM of as-fabricated device; CL signal from hBN (PDF)
