# Supplementary material for: Nanoscale Cathodoluminescence and Conductive Mode Scanning Electron Microscopy of van der Waals Heterostructures
Source: ACS Nano. 2023 Jun 15;17(12):11882–91. doi: 10.1021/acsnano.3c03261 (PMC10311587; doi:10.1021/acsnano.3c03261)
Supplement: Supplementary file 1 — nn3c03261_si_001.pdf [file nn3c03261_si_001.pdf]

Supporting Information

Nanoscale Cathodoluminescence and Conductive Mode Scanning Electron Microscopy of van der Waals Heterostructures

Hugh Ramsden[1,2], Soumya Sarkar[1], Yan Wang[1], Yiru Zhu[1], James Kerfoot[2], Evgeny M. Alexeev[2], Takashi Taniguchi[3], Kenji Watanabe[4], Sefaattin Tongay[5], Andrea C. Ferrari[2] and Manish Chhowalla[1*]

[1]*Department of Materials Science and Metallurgy, 27 Charles Babbage Road, University of Cambridge, Cambridge, CB3 0FS, United Kingdom*

[2]*Cambridge Graphene Centre, University of Cambridge, 9 J. J. Thomson Ave., Cambridge, CB3 0FA, UK*

[3]*Center for Materials Nanoarchitectonics, National Institute for Materials Science, 1-1 Namiki, Tsukuba 305-0044, Japan*

[4]*Research Center for Functional Materials, National Institute for Materials Science, 1-1 Namiki, Tsukuba 305-0044, Japan*

[5]*School for Engineering of Matter, Transport and Energy, Arizona State University, Tempe, Arizona 85287, United States*

[*]Corresponding author (mc209@cam.ac.uk)

S1. Role of hBN Encapsulation in Obtaining a Bright CL Signal

In Fig.S1 we compare the CL signal obtained from unencapsulated WSe_2 on SiO_2. From this we find no CL signal from WSe_2 can be detected without encapsulation.

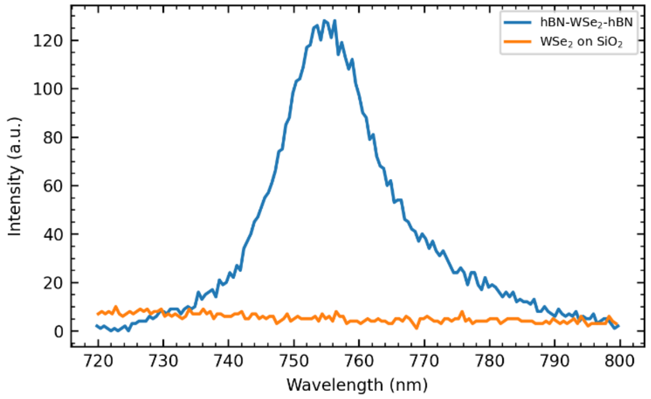

Fig. S1: Lack of CL signal from unencapsulated WSe_2. *Comparison of CL signal from unencapsulated WSe_2 on SiO_2 and a typical encapsulated CL signal. Both measurements taken at 6 keV with a 1 nA beam current and 100 ms acquisition time.*

S2. Device Characterisation

The van der Waals heterostructures (vdW-HSs) are characterized by Raman and PL spectroscopy using a Horiba LabRAM Evolution at 514nm with a 100x objective (NA: 0.9), acquired using, respectively, 1800 l/mm and 150 l/mm gratings. The laser power is <1 mW to exclude heating effects. Fig.S2a indicates that 1L-WSe_2, exhibits E^1_{2g} and A_{1g} modes[1] as a single peak ~250 cm^{-1}. Fig.S2b shows the Raman spectra of hBN encapsulated few layer graphene (FLG), both with and without the overlying 1L-WSe_2, with the G peak ~1580 cm^{-1} and structured 2D band, as expected for FLG.[2] The feature seen in Fig.1b~1366 cm^{-1} corresponds to the E_{2g} mode of hBN.[3] This demonstrates all constituent layers within the vdW-HS are preserved throughout the fabrication process. From Fig. S2a, we estimate the strain variation across the 1L-WSe_2 by comparing the position of the degenerate $E^1_{2g} + A_{1g}$ peak and the 2LA(M) overtone peak, as extracted by Lorentzian fitting, at different points.[4] We estimate a tensile strain variation ~0.13% across the hBN encapsulated 1L-WSe_2 based upon the shift ($\Delta \sim 0.07$ cm^{-1}) of the 2LA (M) Raman mode.[4]

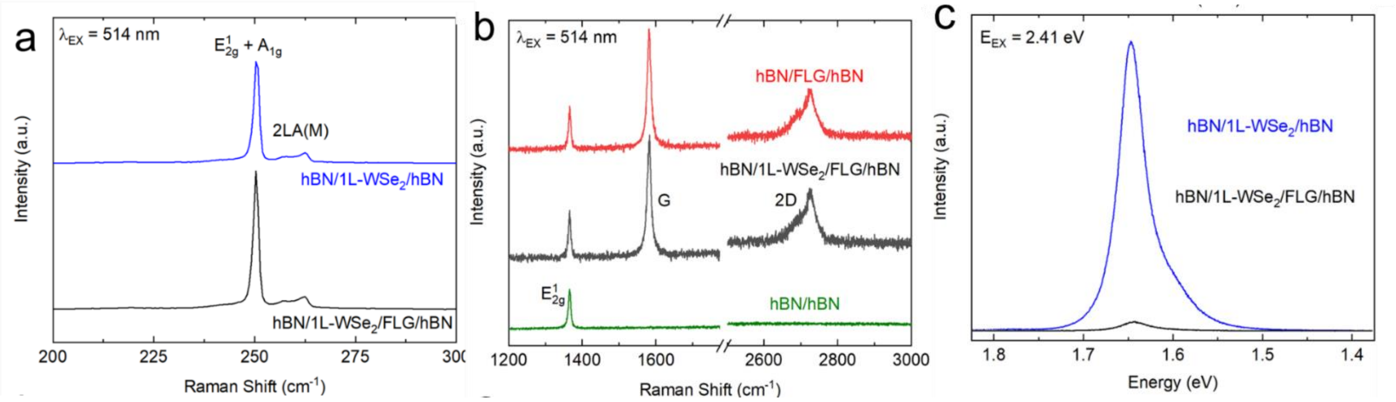

Fig. S2: Raman and PL of the vdW-HS. a) Raman spectra of hBN/1L-WSe_2/hBN (blue) and hBN/1L-WSe_2/FLG/hBN (black) on 285nm SiO_2/Si. b) Raman measurements of hBN/1L-WSe_2/FLG/hBN (black), hBN/FLG/hBN (red) and the surrounding hBN of the same thickness (green). All Raman spectra are normalized to the intensity of the Raman peak of the underlying Si at 520 cm^{-1}. c) PL spectra of hBN/1L-WSe_2/hBN (blue) and partially quenched hBN/1L-WSe_2/FLG/hBN (black).

PL measurements on 1L-WSe_2 within the vdW-HS in the absence of FLG confirm that the flake is 1L-WSe_2, Fig.S2c. The peak~750 nm is ascribed to the neutral exciton, X^0.[1] The relative intensity and full width at half maximum (FWHM) of both hBN/1L-WSe_2/FLG/hBN and hBN/1L-WSe_2/ hBN on 285

nm SiO_2/Si are extracted *via* Lorentzian fitting: FWHM~14.8 nm (0.016 eV) and 16.5 nm (0.018 eV), respectively. The relative emission intensity of 1L-WSe_2 in contact with FLG within the vdW-HS significantly decreases due to exciton dissociation induced by interlayer charge transfer.[5] The absolute value varies spatially, with a minimum reduction factor ~1100.

S3. Comparison of Spatial Resolution of RCI and CL

Fig.S3 plots the edge response in CL and electrode current along the line indicated in Fig.2c. Comparing the edge response in CL from 1L-WSe_2 to the electrode current we can see they do not perfectly overlap. The electrode current signal decays slower. This is because the spatial resolution of CL is high[6], and is limited by the diffusion of excitons in the hBN[6], however RCI tracks majority carriers, which are free to travel through the hBN. The decay in signal can be understood by considering what happens as the probe is scanned beyond the edge of the grounded 1L-WSe_2. Away from the grounded 1L-WSe_2, electrons can either travel horizontally through hBN back to the 1L-WSe_2, or vertically down to the substrate. The distance through the hBN to the substrate will remain constant, whereas, as the horizontal distance increases, the resistance of the path through hBN to the electrode also increases, leading to a drop in electrode current.

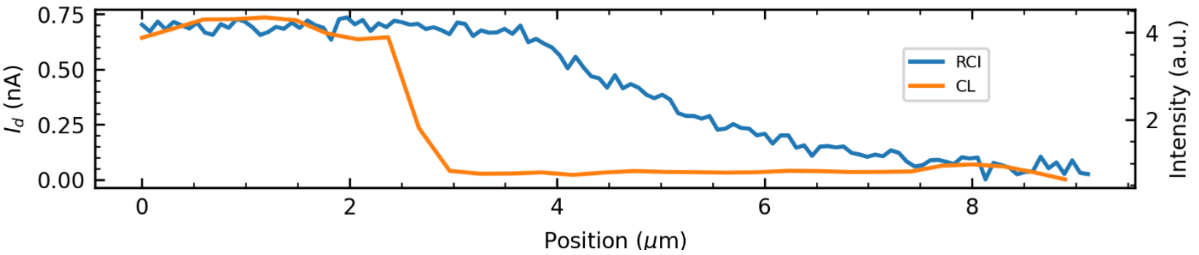

Fig. S3: Edge response of CL and RCI signals along the path indicated by green arrow in Fig.2c. The CL line profile plots the integrated intensity of the 1L-WSe_2 peak vs position along the path. The orange line plots current measured via FLG electrode, I_e, vs position along the path. The black dotted line indicates the approximate edge of 1L-WSe_2, hence, the edge response of the CL signal is sharper than the RCI signal.

S4. Beam Induced Heating of Sample

Ref.[7] studied the heating of samples under electron irradiation, finding:

$$\Delta T \approx \frac{0.099 \cdot VI}{\sqrt{\pi} rk}$$

Where ΔT is the beam induced temperature increase, V is the acceleration voltage, I is the beam current, r is the width of the beam and k is the material thermal conductivity. For our samples, we estimate an upper limit on beam induced heating using the values in Table S1.

Table S1: Parameters used to estimate beam induced heating.

Parameter	Value
V	6000 V
I	1 nA
r	5 nm
K	4 W m^{-1} K^{-1} [[8]]
T_0	300 K

This estimates the beam heating to result in up to a 53 K increase in sample temperature. Given that the sample is kept at 300 K, this would raise the temperature to 353K. Additionally, previous works have reported that the temperature of the exciton population during cathodoluminescence can be larger than the lattice, in contrast to photoluminescence.[9]

S5 Discussion of CL Line-shape

The asymmetry of luminescence from WSe_2 is often attributed a trion component, found at ~30 meV below the neutral exciton.[10–13] The intensity and energy of luminescence from trions has been shown to be sensitive to the carrier concentration in WSe_2.[11–13] Therefore some changes to the CL line-shape under electron beam doping seen in Figure 3e may be expected. At present, studies of emission from

trions at room temperature are lacking. To confirm if the carrier concentration can significantly affect the luminescence line-shape under the conditions used in this work, in Fig.S4a, we collect PL spectra whilst using back gating to modulate the carrier concentration. In Fig.S4a, we see that applying a bias of 30 V, the luminescence intensity reduces by 25% - a comparable amount to that seen after 28s of irradiation at 6 keV. In Fig.S4b we see that despite the reduction in luminescence intensity and change in carrier density, the line-shape of the luminescence is unchanged. This is in agreement with the charging dependent CL spectra shown in (Fig.3e).

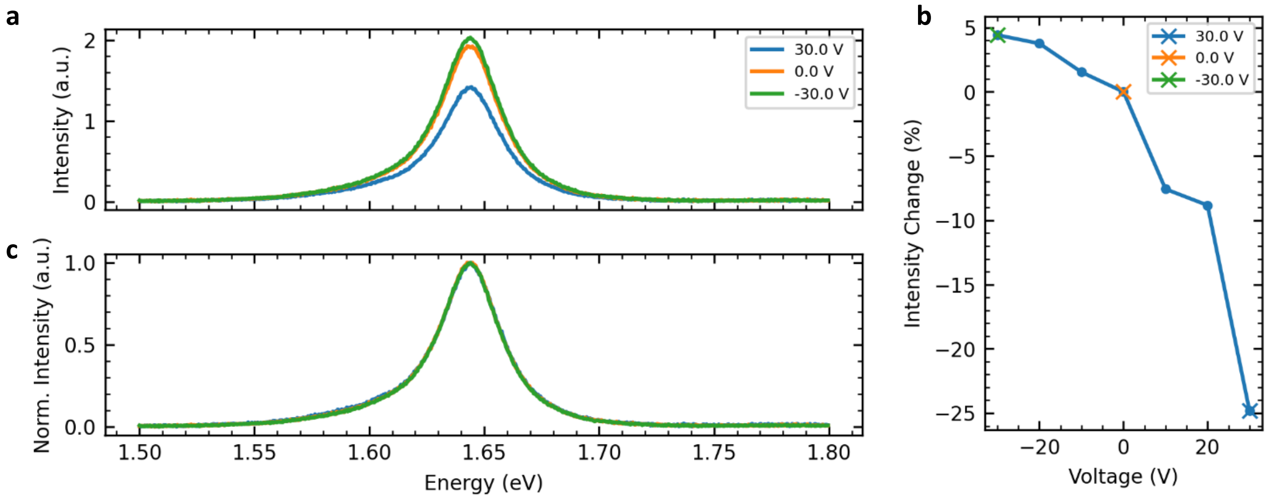

Fig. S4: Gate dependent PL measurements. a) Room temperature gate dependent PL spectra, measured at a fixed position on hBN encapsulated WSe_2 sample. b) Change in integrated intensity from 0 V measurement verses applied voltage. c) Normalised PL spectra from (a).

S6. Assessing Influence of Dielectric Environment on Emission Energy

As described in the main text, a number of potential factors can influence the luminescence energy of 1L-TMDs, including temperature[10,14], local dielectric environment[15,16] and strain.[17,18] However, in the case of the data in Fig.1e, we attribute the variation observed to strain. We eliminate temperature as the beam parameters are fixed, hence this should result in a uniform temperature over the sample.

To assess the role of dielectric environment we note that sources of dielectric heterogeneities such as bubbles and contaminants have been shown to quench emission.[19] Therefore, were dielectric environment to be the dominant factor determining CL energy, regions with lower intensities would exhibit higher energies.[15,16] To check this, in Fig.S5, we plot a scatter of peak energies versus peak intensity. With this we observe no clear correlation between the peak energy and intensity, suggesting dielectric environment is not a significant factor dictating the emission energy. We believe this may be because fitting can only be performed in regions with sufficient intensity (> 2400 counts), which may eliminate regions with bubbles and contaminants from this analysis.

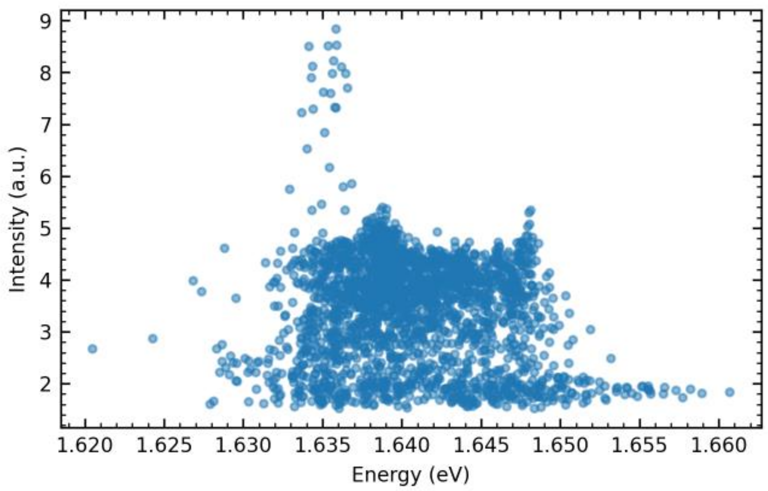

Fig. S5: **Assessing influence of dielectric environment on emission energy.** Scatter plot of integrated intensity verses emission energy from fitted data from Fig.1e.

S7. Band Alignment of vdW-HS

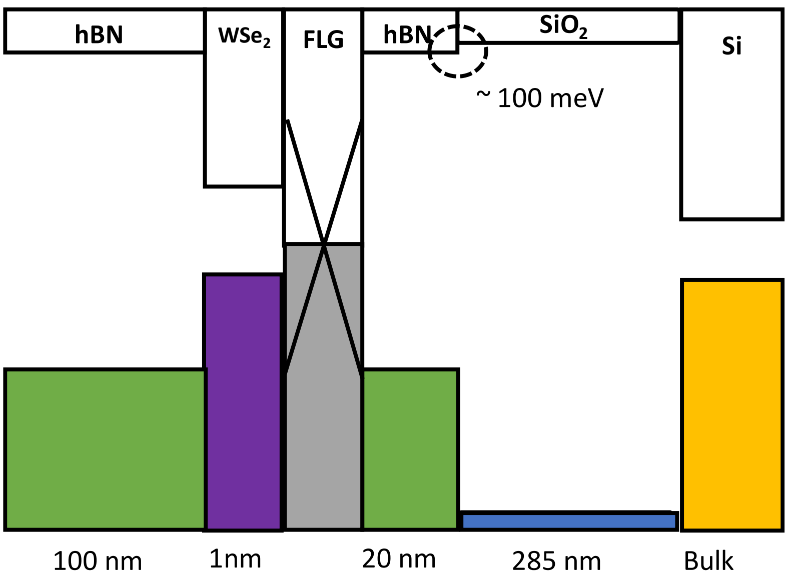

Fig. S6: Band alignment for vdW-HS used in this work. The conduction bands of hBN and SiO_2 are offset by 100 meV, hence charge cannot transfer into the SiO_2. Values for electron affinity and band gaps for hBN taken from Ref. [20], for 1L-WSe_2 Ref. [21], FLG, Ref. [22], Si and SiO_2 Ref. [23].

S8. Monte-Carlo Simulations

The Nebula Monte-Carlo software allows a number of electron cascades to be simulated[24]. Each cascade is independent.[24] Through a compiler switch, the generation of secondary electrons during the cascade can be turned on and off. With secondary electrons off, one only studies the path of primary or beam electrons (denoted with a superscript *pri*). With secondary electrons on, one also studies the generation of secondary electrons (denoted with superscript *all*).

A 1000 X 1000 X 10,000 nm (x, y, z) total sample size is used, with a virtual detector placed 10 nm above the sample, allowing any electrons that leave the sample surface to be counted. Ideal mirrors are placed on the sides of the simulation volume and a terminator placed at the bottom surface. The simulation volume was selected to be beyond the interaction volume of all beams simulated to avoid influencing results.

The backscattered electron coefficient η is defined as the ratio of backscattered electrons to incident electrons.[25] To estimate this, we count the number of electrons detected whilst secondary electron generation is off N_{det}^{pri} and divide by the number of beam electrons N_{beam}, giving $\eta = N_{det}^{pri}/N_{beam}$. The secondary electron coefficient δ is defined as the ratio of secondary electrons to incident electrons.[25] As, Nebula cannot discriminate between detection events from secondary or primary electrons[24], to calculate the secondary electron coefficient δ, we take the number of detection events with secondary electrons turned on N_{det}^{all} and subtract the number of detection events without N_{det}^{pri}, obtaining $\delta = (N_{det}^{all} - N_{det}^{pri})/N_{beam}$.

To obtain the depth profile of primary electrons we examine a cascade with no secondary electron generation. Some primary electrons will be backscattered and will hit the virtual detector. Therefore we ignore any electron collisions for electrons that are detected and create a histogram of the depths of the final collision event for undetected primary electrons.

S9. Estimation of Net Charge Deposited into vdW-HS through Monte-Carlo Simulations

To understand the deposition of electrons into vdW-HS in further detail, we consider the range of possible electron-sample interactions and how these may give rise to charging in samples. Fig.S7a shows a range of electron-sample interactions[26,27]. Per unit time, the net charge deposited in a sample under electron irradiation can be expressed by $I_b' = I_b[1 - (\eta + \delta)]$[26–28]. The thickness of our vdW-HS is ~245 nm. This is of the same order or lower than the electron penetration. Since we only consider electrons deposited into our vdW-HS, we must account for this. To do so, we introduce the parameter Y, which represents the fraction of electrons deposited within the vdW-HS thickness. As secondary electron emission only takes place from the top few-nm[27], the current deposited into a vdW-HS device is: $I_d = I_b[Y(1 - \eta) - \delta]$.

Table S2: Material parameters used for hBN:

Parameter	Value	Reference
**Density**	2.1 g cm^{-3}	[29]
**Valence band width**	6.87 eV	[20]
**Band gap**	5.99 eV	[20]
**Dielectric function**	(Available upon request)	[30,31]
**Electron affinity**	0.86 eV	[20]
**Lattice constant**	2.51 Å	[32]
**Electron effective mass**	2.2 m_e	[32]
**Speed of sound**	19.4 kms^{-1}	[33]
**Acoustic deformation potential**	3.66 eV	[33]

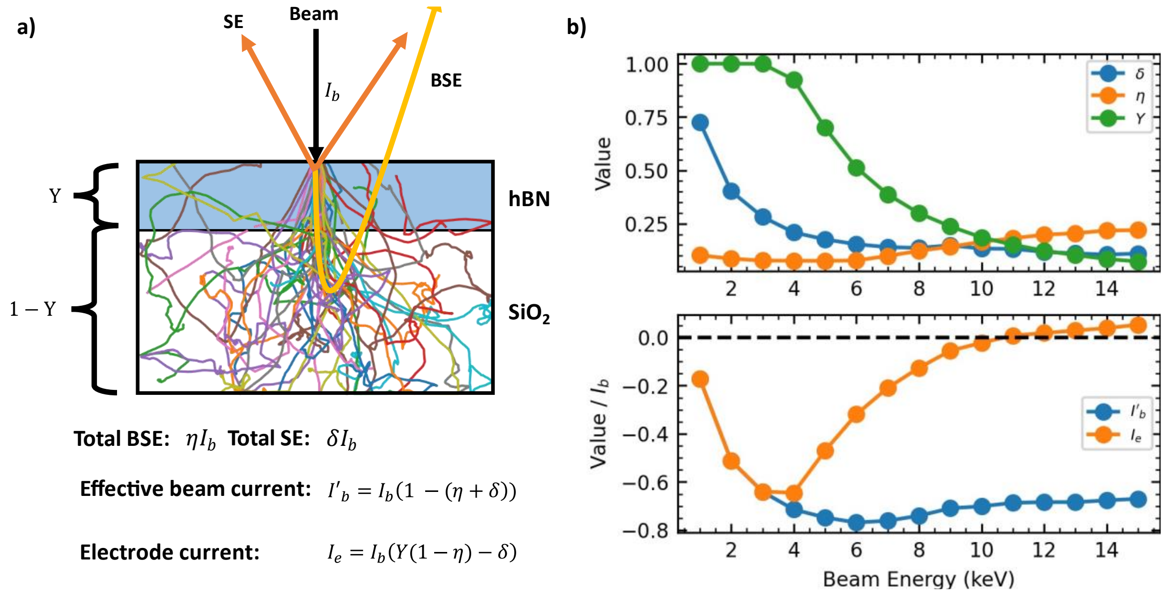

Fig. S7: a) Range of electron-sample interactions and key parameters. The electron beam impinges on the sample I_b. A certain fraction η of those electrons are backscattered. For each impinging electron, a certain fraction δ of secondary electrons leaves the sample's surface. A fraction Y of electrons that come to rest in the vdW-HS . The degree of charging of the vdW-HS can be predicted using $I_d = I_b[Y(1-\eta) - \delta]$ b) Monte-Carlo estimates for for η, δ, Y as a function of acceleration voltage for 1L-WSe_2 encapsulated in 235 nm hBN on top and 10 nm on the bottom. At ~10 keV, the net charge on the vdW-HS is in equilibrium. > 11 keV, Y is sufficiently small that any negative charging from deposited electrons is outweighed by secondary electron emission. < 7 keV, Y climbs rapidly, resulting in strong negative charging.

Fig.S7b uses Monte-Carlo simulations to estimate these parameters for our hBN/1L-WSe_2/hBN on SiO_2, over a range of beam acceleration voltages used in SEM-CL. For voltages > 4keV, both η and δ are not strongly dependent on voltage, instead the most significant factor is Y. For lower voltages, since the penetration depth is comparable to the vdW-HS thickness, most electrons are deposited into the sample, leading to negative charging at > 20% of the beam current. However, as the voltage increases, a threshold is reached where the number of electrons deposited in the vdW-HS drops off sufficiently that the secondary electron emission from the sample surface starts to dominate, giving rise to net positive charging. For very low voltages (< 2keV), the secondary electron coefficient rises significantly, resulting in a decrease in negative charging. Similar results were reported for irradiation of insulating thin films.[27,34]

Fig.S8 plots Y and I_b' over a range of hBN thicknesses.

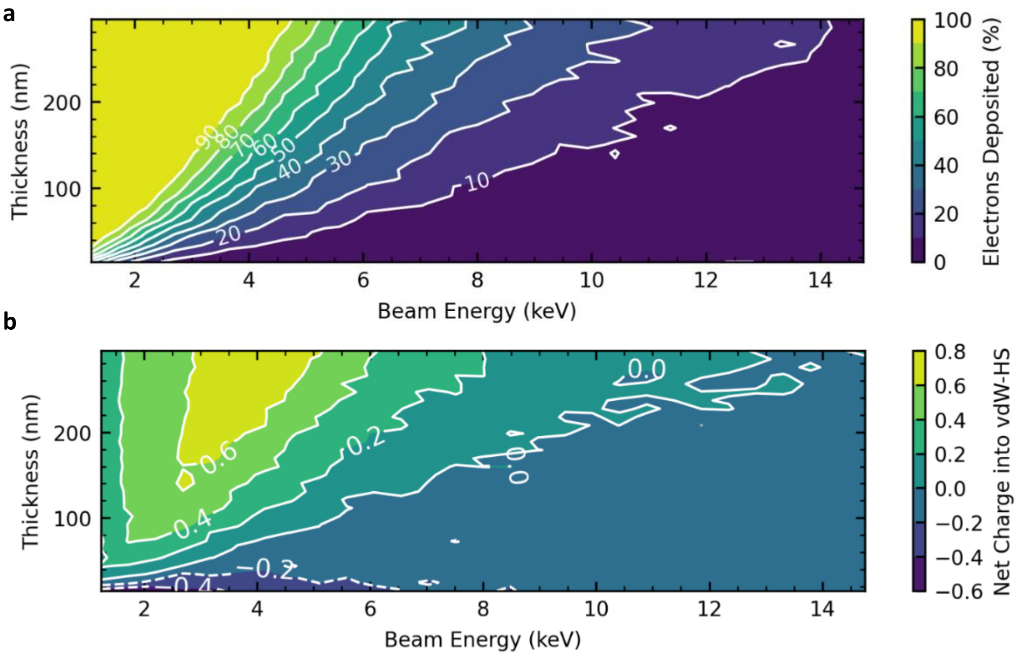

Fig. S8: a) Map of electron fraction deposited over a range of sample thicknesses and acceleration voltages. Increasing the sample thickness or decreasing the acceleration voltage increase the number of carriers deposited into the sample. This can be tuned by adjusting the acceleration voltage. b) Map of net charge within vdW-HS, accounting for emission of secondary and backscattered electrons, as well as electron deposition into the substrate.

S10. AFM of Device

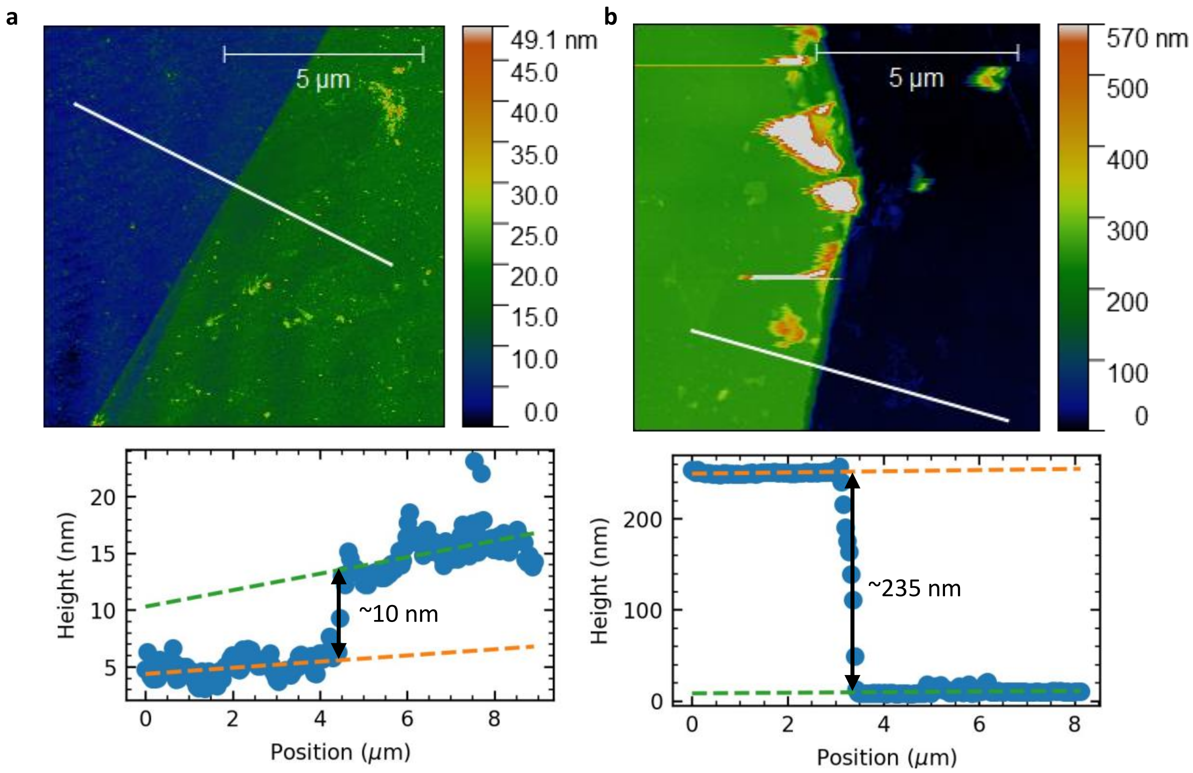

Fig. S9: AFM of device. a) AFM of bottom hBN. Top: AFM image of sample height over step between substrate and bottom hBN. Bottom: Height profile used to determine bottom hBN thickness~10 nm[check]. Profile indicated by white line in Top. b) AFM of top hBN. Top: AFM image of sample height over step between substrate and top hBN. Bottom: Height profile used to determine top hBN thickness~235 nm. Profile indicated by white line in Top.

S11. CL Signal from hBN

CL emission also occurs from hBN.[35–38] In Fig.S10 a CL spectrum measured in regions with encapsulated WSe_2 and encapsulated FLG. In these spectra we see weak luminescence between 300-700 nm which we attribute to defect emission from the hBN[36–39]. In the spectral range including WSe_2 there is no background from the hBN.

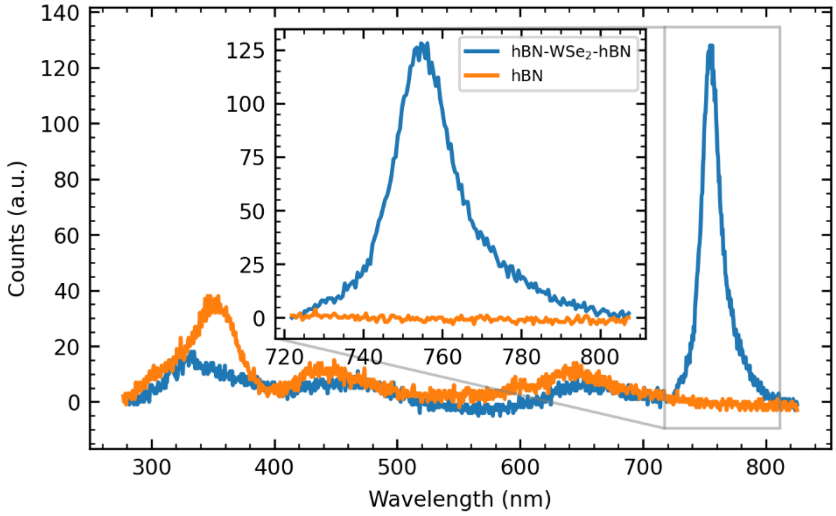

Fig. S10: CL signal from hBN. Comparison of CL spectra from hBN encapsulated FLG and hBN encapsulated FLG. Inset shows spectral region where emission from WSe_2 is dominant.

References

1. Tonndorf, P. *et al.* Photoluminescence emission and Raman response of monolayer MoS_2, $MoSe_2$, and WSe_2. *Opt. Express, OE* **21**, 4908–4916 (2013).
2. Ferrari, A. C. *et al.* Raman Spectrum of Graphene and Graphene Layers. *Phys. Rev. Lett.* **97**, 187401 (2006).
3. Reich, S. *et al.* Resonant Raman scattering in cubic and hexagonal boron nitride. *Phys. Rev. B* **71**, 205201 (2005).
4. Dadgar, A. M. *et al.* Strain Engineering and Raman Spectroscopy of Monolayer Transition Metal Dichalcogenides. *Chem. Mater.* **30**, 5148–5155 (2018).
5. Yang, B. *et al.* Effect of Distance on Photoluminescence Quenching and Proximity-Induced Spin–Orbit Coupling in Graphene/WSe_2 Heterostructures. *Nano Lett.* **18**, 3580–3585 (2018).
6. Francaviglia, L. *et al.* Optimizing cathodoluminescence microscopy of buried interfaces through nanoscale heterostructure design. *Nanoscale* 10.1039.D1NR08082B (2022) doi:10.1039/D1NR08082B.

7. Vine, J. & Einstein, P. A. Heating effect of an electron beam impinging on a solid surface, allowing for penetration. *Proc. Inst. Electr. Eng. UK* **111**, 921 (1964).
8. Jiang, P., Qian, X., Yang, R. & Lindsay, L. Anisotropic thermal transport in bulk hexagonal boron nitride. *Phys. Rev. Materials* **2**, 064005 (2018).
9. Radford, C. J., Hagston, W. E. & Bryant, F. J. Free exciton motion and exciton-phonon interaction studies using cathodoluminescence. *Journal of Luminescence* **5**, 47–56 (1972).
10. Huang, J., Hoang, T. B. & Mikkelsen, M. H. Probing the origin of excitonic states in monolayer WSe_2. *Scientific Reports* **6**, 22414 (2016).
11. Ross, J. S. *et al.* Electrically tunable excitonic light-emitting diodes based on monolayer WSe_2 p–n junctions. *Nature Nanotech* **9**, 268–272 (2014).
12. Li, Z. *et al.* Revealing the biexciton and trion-exciton complexes in BN encapsulated WSe_2. *Nat Commun* **9**, 3719 (2018).
13. Wang, J. *et al.* Polarity Tunable Trionic Electroluminescence in Monolayer WSe_2. *Nano Lett.* **19**, 7470–7475 (2019).
14. Yan, T., Qiao, X., Liu, X., Tan, P. & Zhang, X. Photoluminescence properties and exciton dynamics in monolayer WSe_2. *Appl. Phys. Lett.* **105**, 101901 (2014).
15. Stier, A. V., Wilson, N. P., Clark, G., Xu, X. & Crooker, S. A. Probing the Influence of Dielectric Environment on Excitons in Monolayer WSe_2: Insight from High Magnetic Fields. *Nano Lett.* **16**, 7054–7060 (2016).
16. Li, Z. *et al.* Dielectric Engineering for Manipulating Exciton Transport in Semiconductor Monolayers. *Nano Lett.* **21**, 8409–8417 (2021).
17. Frisenda, R. *et al.* Biaxial strain tuning of the optical properties of single-layer transition metal dichalcogenides. *npj 2D Materials and Applications* **1**, 1–7 (2017).
18. Aslan, B., Deng, M. & Heinz, T. F. Strain tuning of excitons in monolayer WSe_2. *Phys. Rev. B* **98**, 115308 (2018).
19. Nayak, G. *et al.* Cathodoluminescence enhancement and quenching in type-I van der Waals heterostructures: Cleanliness of the interfaces and defect creation. *Phys. Rev. Materials* **3**, 114001 (2019).

20. Wickramaratne, D., Weston, L. & Van de Walle, C. G. Monolayer to Bulk Properties of Hexagonal Boron Nitride. *J. Phys. Chem. C* **122**, 25524–25529 (2018).

21. Schulman, D. S., Arnold, A. J. & Das, S. Contact engineering for 2D materials and devices. *Chem. Soc. Rev.* **47**, 3037–3058 (2018).

22. Yan, R. *et al.* Determination of graphene work function and graphene-insulator-semiconductor band alignment by internal photoemission spectroscopy. *Appl. Phys. Lett.* **101**, 022105 (2012).

23. Fujimura, N., Ohta, A., Makihara, K. & Miyazaki, S. Evaluation of valence band top and electron affinity of SiO_2 and Si-based semiconductors using X-ray photoelectron spectroscopy. *Jpn. J. Appl. Phys.* **55**, 08PC06 (2016).

24. van Kessel, L. & Hagen, C. W. Nebula: Monte Carlo simulator of electron–matter interaction. *SoftwareX* **12**, 100605 (2020).

25. Reimer, L. Chapter 4. Emission of Backscattered and Secondary Electrons. in *Scanning electron microscopy: physics of image formation and microanalysis / Ludwig Reimer.* (Springer, 1998).

26. Reimer, L. Chapter 3. Electron Scattering and Diffusion. in *Scanning electron microscopy: physics of image formation and microanalysis / Ludwig Reimer.* (Springer, 1998).

27. Joy, D. C. & Joy, C. S. Low voltage scanning electron microscopy. *Micron* **27**, 247–263 (1996).

28. Holt, D. B. *Quantitative scanning electron microscopy / edited by D. B. Holt ... [et al.].* (Academic Press, 1974).

29. Madelung, O. & Madelung, O. *Semiconductors: Group IV Elements and III-V Compounds*. (Springer Berlin / Heidelberg, 1991).

30. Nicholls, R. J. *et al.* Low-loss EELS of 2D boron nitride. *J. Phys.: Conf. Ser.* **371**, 012060 (2012).

31. Laturia, A., Van de Put, M. L. & Vandenberghe, W. G. Dielectric properties of hexagonal boron nitride and transition metal dichalcogenides: from monolayer to bulk. *npj 2D Mater Appl* **2**, 1–7 (2018).

32. Knobloch, T. *et al.* The performance limits of hexagonal boron nitride as an insulator for scaled CMOS devices based on two-dimensional materials. *Nat Electron* **4**, 98–108 (2021).

33. Bruzzone, S. & Fiori, G. Ab-initio simulations of deformation potentials and electron mobility in chemically modified graphene and two-dimensional hexagonal boron-nitride. *Appl. Phys. Lett.* **99**, 222108 (2011).

34. Cazaux, J. Some considerations on the electric field induced in insulators by electron bombardment. *Journal of Applied Physics* **59**, 1418–1430 (1986).

35. Taniguchi, T. & Watanabe, K. Synthesis of high-purity boron nitride single crystals under high pressure by using Ba–BN solvent. *Journal of Crystal Growth* **303**, 525–529 (2007).

36. Bourrellier, R. *et al.* Bright UV Single Photon Emission at Point Defects in h-BN. *Nano Lett.* **16**, 4317–4321 (2016).

37. Museur, L., Feldbach, E. & Kanaev, A. Defect-related photoluminescence of hexagonal boron nitride. *Phys. Rev. B* **78**, 155204 (2008).

38. Mendelson, N. *et al.* Identifying carbon as the source of visible single-photon emission from hexagonal boron nitride. *Nat. Mater.* **20**, 321–328 (2021).

39. Hayee, F. *et al.* Revealing multiple classes of stable quantum emitters in hexagonal boron nitride with correlated optical and electron microscopy. *Nat. Mater.* **19**, 534–539 (2020).
